# Situational judgement tests: important clarifications regarding the methodology

**DOI:** 10.15694/mep.2017.000219

**Published:** 2017-12-06

**Authors:** Rebecca Beesley, Angelica Sharma, Jason Leo Walsh, Benjamin Howell Lole Harris, David John Wilson

**Affiliations:** 1 Cardiff University School of Medicine; 2 Saint George's Healthcare NHS Trust; 3 University Hospital of Wales

**Keywords:** Situational judgement test, Selection

## Abstract

This article was migrated. The article was marked as recommended.

## Letter

Editor,

We thank the Editor of *Medical Teacher* for suggesting that we continue the discussion through *MedEdPublish.*

In our previous account, we asked the question: who knows the right answer when dealing with the ranking format questions in a situational judgement test (SJT)?. ^1^ The results suggested that the discriminatory ability of ranking questions may be driven by ranking options of medium/lower appropriateness, and the clinical relevance of this was questioned. To improve clinical pertinence, a single-best answer format was suggested.

We appreciate the clarification provided Patterson et al ^2^ in their response to our suggestions, particularly on the matter of which subject matter experts (SMEs) are utilised at the concordance stage. In view of their comments, reanalysis of our data, retaining only ‘senior doctors who work closely with FY1 doctors’ (n = 49, approximately 5-7 times larger than the standard sample of SMEs), the results continue to show concerning trends.

In only 4 out of 10 (40%) of the questions in our mock SJT, our larger sample of SMEs identified the original answer key as being “correct”. Here, we show a summary of all ten tested questions to clarify the extent of disagreement between our SMEs and the “correct (pre-determined) answers”.

**Table 1.** Summary of independent SME responses (n = 49) to a mock SJT created by randomly selecting ten ranking format questions from the online practise SJT available on the UK Foundation Programme Office website (2016). Where there was is disagreement between SMEs and the “correct answer” the row is emboldened. Obelisks denote worrying disagreements.

“Correct” (pre-determined) answer	Frequency count (n=49)	Most frequent answer rank sequence	Frequency count (n=49)	Popularity of “correct” answer order in our sample
**53214 ^†^**	**4**	**43125 ^†^**	**6**	**Joint 3 ^rd^ (with two other sequences)**
**34215 ^†^**	**4**	**35214 ^†^**	**6**	**Joint 3 ^th^ (with one other sequence)**
**54231**	**2**	**54321**	**6**	**Joint 5 ^th^ (with four other sequences)**
**21543**	**2**	**21534**	**11**	**Joint 5 ^th^ (with three other sequences)**
**21354**	**7**	**21345**	**19**	**Joint 2 ^nd^ (with one other sequence)**
**13452**	**9**	**13542**	**16**	**2 ^nd^**
**12345**	**14**	**12345**	**14**	**1 ^st^**
**51234**	**31**	**51234**	**31**	**1 ^st^**
**51342**	**12**	**51342**	**12**	**1 ^st^**
**15432**	**15**	**15432**	**15**	**1 ^st^**

**Figure 1.**
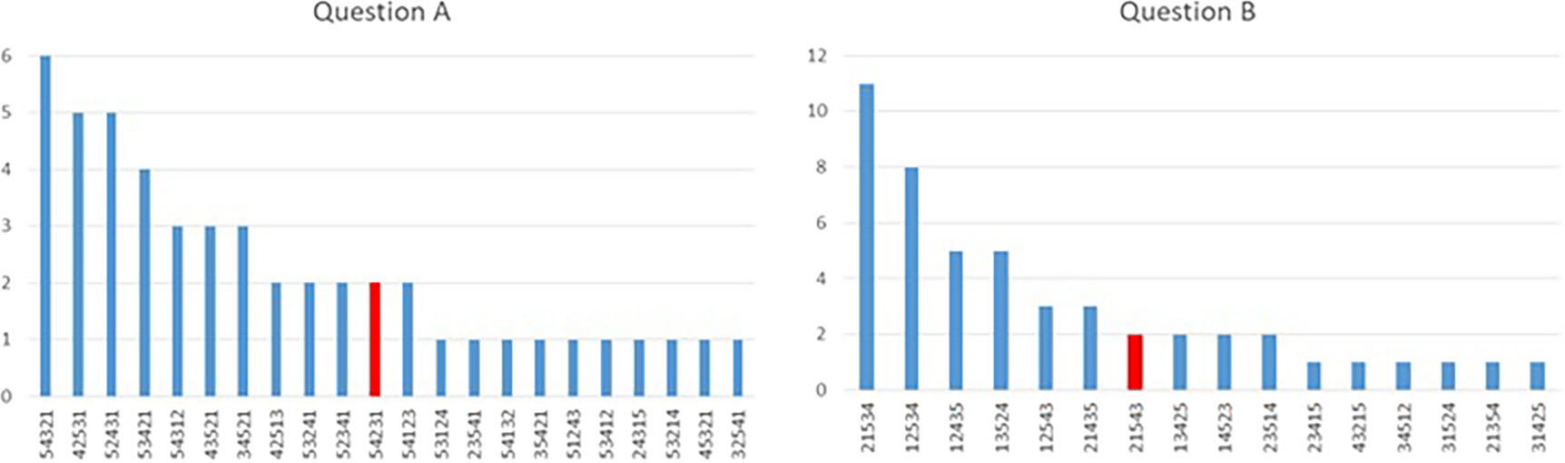
Bar charts highlighting the heterogeneity of answers between SMEs in two SJT ranking questions from the online practise SJT available on the UK Foundation Programme Office website (2016). “Correct” (pre-determined) answers are marked in red.

There appears to be a marked disagreement over the “correct answer” and what SMEs perceive as the correct answer ( Table 1 and Figure 1). The heterogeneity of SME responses underlines the subjectivity of SJT ranking questions. Even if SME unanimous agreement is not sought, one would hope it was common.

Notably, when looking at the most frequent answers in our sample, in one question, the *most inappropriate* option was chosen as the *most appropriate* option and vice versa. In another, the *most inappropriate* option was chosen as the *second most appropriate* option. These two questions are denoted with obelisks in Table 1. Furthermore, over half the SMEs arrived at the “correct” sequence in only one of the 10 questions.

Near-miss scoring is all well and good, but it is unlikely to correct for the subjectivity of the questions and is likely to contribute to the volatility of the Foundation Programme SJT, where student scores tend to cluster close to the mean. ^3^

Our study highlights that there is uncertainty surrounding the “correct answer” in ranking questions within the Foundation Programme Situational Judgement Test. We feel this uncertainty needs further investigation. We would encourage independent scrutiny of the concordance data between SMEs and the itemised pilot data to ensure the determined “correct answer” is free from uncertainty. Independent post-hoc analysis of the candidate ranking sequences would be very interesting to examine candidate answer heterogeneity.

The SJT ranking format is claimed to be less susceptible to coaching effects than single-best answer questions. Is this simply because it is hard to find consensus over what is truly the correct answer?

## Notes On Contributors

Rebecca Beesley and Angelica Sharma are senior medical school students and were joint authors on this article.

Benjamin Harris and Jason Walsh are practising doctors currently in higher training.

David Wilson is a professor of medical education and the Director of Admissions for the medical school.
