# Supplementary material for: Situational judgement tests: important clarifications regarding the methodology
Source: MedEdPublish (2016). 2017 Dec 6;6:219. [Version 1] doi: 10.15694/mep.2017.000219 (PMC10885230; doi:10.15694/mep.2017.000219)
Supplement: Peer review history

# Reviewer report: Reviewer response for version 1

**DOI:** 10.21956/mep.19405.r28469

**Published:** 2019-04-21

**Authors:** Ken Masters

**Affiliations:** 1 Sultan Qaboos University

This review has been migrated. The reviewer awarded 4 stars out of 5 A short, sharp, and very well-focused letter questioning the validity of the ranking format of questions in situational judgement test. The authors end with the rather provocative question: “The SJT ranking format is claimed to be less susceptible to coaching effects than single-best answer questions. Is this simply because it is hard to find consensus over what is truly the correct answer?”Although the sample of questions is small, it demonstrates the problem perfectly. Even if those were the only questions in the entire test that had these problems, it would be worrying.A good read, and enough to, hopefully, prompt a serious review of the test.

Reviewer Expertise:

NA

No decision status is available

# Reviewer report: Reviewer response for version 1

**DOI:** 10.21956/mep.19405.r28470

**Published:** 2018-06-22

**Authors:** Trevor Gibbs

**Affiliations:** 1 AMEE

This review has been migrated. The reviewer awarded 5 stars out of 5 This is becoming an interesting discussion about a now well -used methodology. If there is anything that we need to remove from any sort of assessment it is subjectivity and inter-rater variability. Certainly the other main issue is uncertainty regarding the accepted answer and this is what the discussion is about. I feel that the authors are correct in asking this question in the initial paper and this response letter continues to support that we still are not that closer to the answer. I eagerly look forward to a continuance of this discussion and I would suggest that all those involved in SJTs should be too.

Reviewer Expertise:

NA

No decision status is available
